# Major Adverse Cardiac and Cerebrovascular Events in Patients Undergoing Simultaneous Heart Surgery and Carotid Endarterectomy

**DOI:** 10.3390/jcdd10080330

**Published:** 2023-08-02

**Authors:** Stephen Gerfer, Walid Bennour, Alina Chigri, Ahmed Elderia, Ihor Krasivskyi, Clara Großmann, Christopher Gaisendrees, Borko Ivanov, Soi Avgeridou, Kaveh Eghbalzadeh, Parwis Rahmanian, Ferdinand Kuhn-Régnier, Navid Mader, Ilija Djordjevic, Anton Sabashnikov, Thorsten Wahlers

**Affiliations:** Department of Cardiothoracic Surgery, Heart Center, University Hospital of Cologne, 50937 Cologne, Germanyahmed.elderia@uk-koeln.de (A.E.); soi.avgeridou@uk-koeln.de (S.A.);

**Keywords:** open heart surgery, CABG, valve surgery, carotid TEA, carotid endarterectomy

## Abstract

Background. Patients with simultaneous relevant internal carotid artery stenosis and coronary artery heart or valve disease represent a high-risk collective with respect to cerebral or cardiovascular severe events when undergoing surgery. There exist several concepts regarding the timing and modality of carotid revascularization, which are controversially discussed in patients with heart disease. More data regarding outcome predictors and measures are needed to gain a better understanding of the best treatment option of the discussed patient collective. Methods. This single-center study retrospectively analyzed *n* = 111 patients undergoing heart surgery with coronary artery bypass grafting or heart-valve surgery and concomitant carotid surgery due to significant internal carotid artery stenosis. In order to do so, patients were divided into two groups with respect to postoperative major adverse cardiac and cerebrovascular events (MACCE) with thirty-day all-cause mortality, valve related mortality, myocardial infarction, stroke and transitory ischemic attack. Results. Preoperative patient’s characteristic in the no-MACCE and MACCE group were mainly balanced, other than higher rates of chronic obstructive pulmonary disease, chronic kidney disease, instable angina pectoris and prior transitory ischemic attack in the MACCE cohort. The analysis of intraoperative characteristics revealed a higher number of intra-aortic balloon pump implantation, which is in line for a higher number of postoperative supports. Besides MACCE, patients suffered significantly more often from postoperative bleeding events and re-thoracotomy, cardiopulmonary reanimation, new onset postoperative dialysis and prolonged intensive care unit stay related complications. Conclusions. Within the reported patient population suffering from MACCE after a simultaneous carotid endarterectomy and heart surgery, a preoperative history of transitory ischemic attack and kidney disease might account for worse outcomes, as severe events were not only neurologically driven but also associated with postoperative cardiovascular complications following heart surgical procedures.

## 1. Introduction

Coronary artery disease is often associated with central vascular disease as atherosclerotic pathologies might affect several vascular territories concomitantly, including the carotid artery [1,2,3]. In this line, these patients are at a high risk for severe adverse events. Major adverse cardiac and cerebrovascular events (MACCE) within this patient collective are driven by myocardial infarction, stroke or transient ischemic attack (TIA) and an elevated all-cause mortality [4,5,6]. With respect to the optimal approach for patients with concomitant cardiac vitium and internal carotid stenosis, several concepts were analyzed [7,8]. The approach with respect to timing and modality of the carotid revascularization is subject to controversial discussion and should be based on clinical presentation, level of emergency and severity of carotid and coronary artery diseases. Within the current guidelines on myocardial revascularization, carotid revascularization should be considered (Class IIa, Level B) in patients scheduled for coronary artery bypass grafting with a history of stroke or transient ischemic attack within the last 6 months and at least a 50–99% carotid stenosis [9]. In neurologically asymptomatic patients undergoing coronary artery bypass grafting and diagnosed 70–99% carotid stenosis, routinely prophylactic carotid revascularization is not recommended (Class III, Level C); however, it may be considered regarding underlying additional risk factors for stroke (Class IIb, Level C) [9]. As the field of this topic is still controversial, we aimed to analyze patients undergoing simultaneous carotid and coronary revascularization or concomitant heart valve surgery, with respect to MACCE rates and postoperative short-term outcomes to gain a better understanding for outcome predictors.

## 2. Methods

### 2.1. Study Population

This study retrospectively analyzed a cohort of *n* = 111 patients undergoing heart surgery with concomitant carotid surgery due to significant carotid stenosis between 2010 and 2020 with respect to postoperative major adverse cardiac and cerebrovascular events (MACCE) as patients were divided into two groups: MACCE and no-MACCE. All perioperative data were extracted from our institutional database. No ethical approval is required for performing a purely retrospective data analysis regarding standard clinical data without making contact with patients or an external third party.

### 2.2. Variables of Interest

We analyzed patients’ preoperative characteristics before undergoing surgery, as displayed in Table 1. Therefore, we extracted data regarding the patient’s age, sex, height, weight, body mass index and medical history (smoking history, chronic obstructive pulmonary disease, pulmonal hypertension, diabetes mellitus, hyperlipoproteinemia, arterial hypertension, chronic kidney disease, peripheral arterial disease, coronary artery disease, instable angina pectoris, prior myocardial infract, prior coronary intervention, prior percutaneous coronary intervention, shock/decompensation, sinus rhythm, left-ventricular ejection fraction <30% and prior heart surgery). Further, the grade of carotid internal stenosis, number of patients with carotid internal stenosis <90%, bilateral carotid internal stenosis bilateral, other intracranial stenosis, prior carotid internal intervention, neurological disability, prior stroke, prior transitory and ischemic attack were assessed. In addition, the medical therapy of patients was assessed with respect to typical cardiovascular drugs such as Aspirin, P2Y12-Inhibitor, direct oral anticoagulation, Vitamin K-Antagonist, Beta blockers, ACE inhibitor, AT II inhibitor or Statin therapy.

The analysis of intraoperative characteristics for the no-MACCE and MACCE groups is shown in Table 2 with respect to number of carotid endarterectomy using a patch, coronary artery bypass grafting, aortic valve replacement, mitral valve replacement, mitral valve repair, tricuspid valve repair, atrial ablation, atrial exclusion, concomitant surgery, the use of cardio-pulmonal bypass, cardiopulmonary bypass time, aortic cross clamp time, operation time and number of intraoperatively used intra-aortic balloon pump supports.

Table 3 displays postoperative outcome characteristics concerning major adverse cardiac and cerebrovascular events including thirty-day all-cause mortality, valve related mortality, myocardial infarction, stroke and transitory ischemic attack. Further, we investigated outcomes regarding major bleeding, re-thoracotomy, cardio-pulmonary reanimation, shockable rhythm disturbances, new onset postoperative dialysis, intra-aortic balloon pump, extracorporeal membrane oxygenation, brain computer tomography post-operatively, symptomatic transitory psychotic syndrome, serious adverse events other than MACCE, invasive ventilation duration longer than 12 h, intensive care unit stay and overall in-hospital stay.

In general, patients underwent unilateral carotid endarterectomy (CEA) regardless of unilateral or bilateral carotid stenosis with respect to the symptomatic or higher-grade stenosis. All CEAs were performed before initiation of cardio-pulmonary bypass, but after application of the intravenous heparin, the neck incision was closed after antagonization of heparin with protamine. Mostly, the carotid artery was clamped on the proximal and distal site, and a shunt was implanted in the carotid artery as CEA was performed to minimize the ischemia. The decision to use a patch for this procedure was left to the discretion of the attending surgeon. The shunt was removed, and the carotid artery was flushed before closure. Near infrared spectroscopy was used to determine the neurological status of patients during the procedure, as described by our study group before [10].

### 2.3. Statistical Analysis

The statistical analysis was performed with SPSS-Statistics-25 (IBM Corporation, Armonk, New York, NY, USA). The data tables show that all data are given as mean and standard deviation (SD) for continuous variables. Categorical variables are expressed as a percentage (number).

## 3. Results

### 3.1. Preoperative Patients Characteristics

Within the collective of *n* = 111 patients, *n* = 87 patients showed no-MACCE after heart surgery and concomitant CEA, whereas *n* = 24 patients showed at least one MACCE event. The groups were named no-MACCE and MACCE. Baseline characteristics were balanced between groups with respect to age (77 ± 8.3 years vs. 77 ± 7.2 years, *p* = 0.649), male sex (86% vs. 79% *p* = 0.521), height (1.81 ± 0.6 m vs. 1.82 ± 0.6 m, *p* = 0.484), weight (93 ± 4.7 kg vs. 92 ± 5.3 kg, *p* = 0.380) and body mass index (28 ± 1.7 kg/m^2^ vs. 28 ± 1.7 kg/m^2^, *p* = 0.079). The smoking history was also balanced between the no-MACCE and MACCE group (61% vs. 63%, *p* = 0.888), whereas a chronic obstructive pulmonary disease was present statistically more often in the MACCE group (3.4% vs. 17%, *p* = 0.038). Pulmonal hypertension (3.4% vs. 13%, *p* = 0.114) and diabetes mellitus (63% vs. 71%, *p* = 0.489) were comparable between groups, but more patients in the MACCE group showed a history of hyperlipoproteinemia (100% vs. 92%, *p* = 0.045). The incidence of arterial hypertension was balanced (99% vs. 96%, *p* = 0.387), as a higher number of patients in the MACCE group presented with chronic kidney disease (13% vs. 33%, *p* = 0.029). The number of patients with peripheral arterial disease was still comparable (29% vs. 50%, *p* = 0.050), as was the incidence of coronary artery disease (98% vs. 91%, *p* = 0.210) accompanied by a higher rate of patients with an unstable preoperative angina pectoris (5.7% vs. 33%, *p* = 0.001) within the MACCE cohort. Nevertheless, the rate of prior myocardial infraction (80% vs. 13%, *p* = 0.448), prior coronary intervention (10% vs. 25%, *p* = 0.107) and prior percutaneous coronary intervention (9.2% vs. 25%, *p* = 0.098) showed no statistical difference, as were rates for preoperative shock or decompensation (29% vs. 38%, *p* = 0.410). The number of patients with a preoperative left-ventricular ejection fraction less than 30% (3.4 vs. 8.3, *p* = 0.295) was low and balanced, as was the rate of patients with prior heart surgery in both groups (3.4% vs. 4.2, *p* = 1.000). Regarding the grade of internal carotid artery stenosis (87 ± 4.5% vs. 88 ± 3.0%, *p* = 0.353), internal carotid artery stenosis over 90% (51% vs. 38%, *p* = 0.256), bilateral internal carotid artery stenosis (12% vs. 17%, *p* = 0.498), other intracranial internal artery stenosis (1.5% vs. 0.0%, *p* = 0.597), prior carotid internal intervention (4.6% vs. 4.2%, *p* = 1.000), preoperative neurological disability (2.3% vs. 0.0%, *p* = 1.000) and prior stroke (4.6% vs. 13%, *p* = 0.171), no differences between the groups were detected. Within the MACCE cohort, patients suffered significantly more often from a prior transitory ischemic attack (5.7 vs. 21%, *p* = 0.037). The prescription of cardiovascular drugs was balanced with respect to Aspirin (45% vs. 42%, *p* = 0.782), P2Y12-Inhibitor (5.7 vs. 0.0, *p* = 0.583), direct oral anticoagulation (1.1% vs. 0.0%, *p* = 1.000), vitamin K-antagonist (80% vs. 21%, *p* = 0.129), beta blockers (78% vs. 83%, *p* = 0.778), ACE inhibitor (87% vs. 83%, *p* = 0.736), AT II inhibitor (6.9% vs. 17%, *p* = 0.219) and Statin (83% vs. 88%, *p* = 0.759).

### 3.2. Intraoperative Characteristics

The analysis of intraoperative characteristics (Table 2.) of patients undergoing concomitant heart surgery and carotid endarterectomy showed that a patch was used equally in the no-MACCE and MACCE group (16% vs. 13%, *p* = 1.000). The number of procedures with coronary artery bypass grafting (92% vs. 86%, *p* = 0.448), aortic valve replacement (22% vs. 25%, *p* = 0.743, mitral valve replacement (1.1% vs. 8.3%, *p* = 0.117), mitral valve repair (4.6% vs. 4.2%, *p* = 1.000), tricuspid valve repair (2.3% vs. 4.2%, *p* = 0.522), atrial ablation (3.4% vs. 4.2%, *p* = 1.000), atrial exclusion, *n* (5.7% vs. 4.2%, *p* = 1.000) and concomitant heart surgical procedures (20% vs. 29%, *p* = 0.310) were balanced between groups. The need for cardio-pulmonal bypass (92% vs. 92%, *p* = 1.000), the cardiopulmonary bypass time (91 ± 33 min vs. 95 ± 51 min, *p* = 0.543), aortic cross clamp time (52 ± 20 min vs. 51 ± 26 min, *p* = 0.345) and operation time (258 ± 55 min vs. 268 ± 76 min, *p* = 0.659) were balanced between the no-MACCE and MACCE cohort. The initiation of intra-aortic balloon pump support intraoperatively was higher in the MACCE group (1.1% vs. 25%, *p* = 0.002).

### 3.3. Postoperative Outcomes 

Major adverse cardiac and cerebrovascular events were defined as follows (Table 3.): thirty-day all-cause mortality *n* = 7 (29%), valve related mortality *n* = 1 (4.2%), myocardial infarction *n* = 1 (4.2%), stroke *n* = 12 (50%) and transitory ischemic attack *n* = 6 (25%). Table 4 displays the postoperative outcomes of no-MACCE vs. MACCE patients after concomitant heart surgery and carotid endarterectomy. Logically, major adverse cardiac and cerebrovascular events including the above-mentioned parameter occurred significantly more often in the MACCE group: thirty-day all-cause mortality (0.0% vs. 29%, *p* < 0.001), valve related mortality (0.0% vs. 4.2%, *p* = 0.216), myocardial infarction (0.0% vs. 4.2%, *p* = 0.216), stroke (0.0% vs. 50%, *p* < 0.001) and transitory ischemic attack (0.0% vs. 25%, *p* < 0.001). Moreover, other severe events occurred more often within the patient cohort with MACCE as were for major bleeding (0.0% vs. 38%, *p* < 0.001), re-thoracotomy (1.1% vs. 33%, *p* < 0.001), cardiopulmonary reanimation (0.0% vs. 33%, *p* < 0.001), shockable rhythm disturbances (25% vs. 58%, *p* = 0.002), new onset postoperative dialysis (2.3% vs. 29%, *p* < 0.001), intra-aortic balloon pump postoperatively (0.0% vs. 13%, *p* = 0.009), extracorporeal membrane oxygenation (0.0% vs. 4.2%, *p* = 0.216), brain computer tomography post operatively (6.9% vs. 58%, *p* < 0.001), symptomatic transitory psychotic syndrome (39% vs. 50%, *p* = 0.336) and serious adverse events other than MACCE including pneumonia, gastrointestinal bleeding and sepsis (2.3% vs. 17%, *p* = 0.019). The number of patients with invasive ventilation duration longer 12 h was significantly more often in the MACCE group (5.7% vs. 54%, *p* < 0.001) as was the intensive care unit stay (4.1 ± 4.8 days vs. 12 ± 17 days, *p* < 0.001) but not the overall in hospital stay (14 ± 6.5 days vs. 17 ± 17 days, *p* = 0.593).

## 4. Discussion

The main findings of this study are as follows: I. Besides a relatively homogenous distribution of preoperative patients’ characteristics, patients within the MACCE cohort suffered significantly more often from chronic obstructive pulmonary disease, hyperlipoproteinemia, chronic kidney disease, peripheral arterial disease, instable angina pectoris and prior transitory ischemic attack. II. Intraoperative parameters showed no differences regarding type of concomitant surgery or procedure duration, but a higher number of patients with the need for an intraoperatively implanted aortic balloon pump for mechanical support for weaning off the cardiopulmonary bypass or afterwards were identified. III. Major adverse cardiac and cerebrovascular events (MACCE) included thirty-day all-cause mortality, valve related mortality, postoperatively myocardial infarction, stroke and transitory ischemic attack—whereas patients who were allocated to the MACCE group showed at least one of the mentioned symptoms. Other severe adverse events and postoperative complications were significantly associated with MACCE, including prolonged weaning from mechanical ventilation and overall ICU stay.

The incidence of concomitant carotid stenosis and coronary artery disease is not surprising since atherosclerosis is the cause of both diseases and based on the same risk factors as diabetes mellitus, hypertension, smoking or hyperlipidemia [11]. Steinvil et al. showed that existing coronary artery disease or left main coronary artery disease was an independent predictor of severe carotid stenosis or occlusion of the internal carotid artery, as also shown by the high incidence in this study [12]. There remains a controversial discussion regarding the best treatment for patients with concomitant coronary and carotid disease. However, neurological complications in cardiac surgery are multifactorial, as the most severe complication is stroke (cerebral infarction). The usage of a cardiopulmonary bypass with the heart-lung machine and an existing high-grade symptomatic carotid stenosis is predicted as one of the main causes of its etiology. In this context, the perioperative risk of stroke after surgical myocardial revascularization using cardiopulmonary bypass with concomitant untreated carotid stenosis has been described to be up to 14% which often is driven by blood pressure differences [13]. In this line, a high-grade stenosis or occlusion of the carotid artery can cause cerebral infarction hemodynamically due to reduced perfusion pressure within the brain. In general and more commonly, cerebral infarction is caused by thromboembolism by plaque ruptures in the arteries or aorta under clamping of the vessel supplying the brain, which allows these particles to enter the cerebral vasculature and cause embolic occlusion [14]. As the risk of stroke or cerebral infarction increases with the degree of stenosis, patients with high-grade symptomatic carotid stenoses may benefit from revascularization of carotid endarterectomy (CEA) before the initiation of the cardiopulmonary bypass or during cardiac surgery. In doing so, CEA may result in a 17% absolute risk reduction for ipsilateral cerebral infarction over two years in symptomatic patients with stenosis of 70–99% [3,4]. In general, the optimal surgical management of both a higher-grade carotid stenosis and heart disease remains open to debate. However, early isolated thromboendarterectomy results in higher rates of myocardial infarction and mortality [15]. On the other hand, patients who underwent primary coronary artery bypass surgery and subsequent TEA have a higher number of cerebral insults but a lower incidence of myocardial infarction or mortality [15]. The ESC/EACTS guidelines generally do not recommend the “routine prophylactic carotid revascularization” preceding coronary artery bypass grafting (Level of Evidence III, C), while CEA is recommended in case of “one or more features, that may be related with an increased risk of stroke” (Level of Evidence IIb, C) [9]. Further, various studies demonstrated that concomitant heart surgery and CEA procedures can be performed safely with an acceptable risk of neurological complications and mortality [16,17,18,19]. A meta-analysis published by Naylor et al. with *n* = 8972 patients who underwent reverse-staged (CEA after CABG) and simultaneous operations showed that perioperative stroke was the highest in the synchronous cohort, as the mortality rate was the highest in the reverse-staged group with [20]. These findings are in line with recent studies reporting on increased postoperative risks for cardiac events when a staged approach for coronary artery bypass grafting and carotid endarterectomy is performed [6]. Another meta-analysis of observational studies with a total of *n* = 17,469 and *n* = 7552 patients in the combined and staged group, respectively, reported no discrepancies in early postoperative mortality or perioperative stroke and death between the two operative procedures, thereby suggesting comparable outcomes for the discussed approaches in patients with concomitant CEA and coronary artery bypass grafting. A case–control study analyzed patients who underwent combined CEA and coronary artery bypass grafting with respect to a risk-adjusted cohort of patients undergoing an isolated bypass surgery [21]. Within the combined group including *n* = 744 patients, a higher incidence of postoperative complications occurred than in the control group with *n* = 35,539 patients undergoing isolated bypass grafting. Following a risk factor matching, there were no discrepancies regarding stroke rates and all-cause mortality [21]. In line, our reported 30-day all-cause mortality is comparable to the observed mortality rates in the literature for patients undergoing combined CEA and heart surgery, as were rates for postoperative stroke [15,22]. As a comparison, Franchin et al. reported results of a multicenter study which investigated the concomitant CEA and heart surgery with either valve surgery or coronary artery bypass surgery by retrospectively analyzing short-term outcomes of *n* = 386 patients. Within this study, the authors reported on a low rate of neurological events of 2.6% (including 1.3% transient ischemic attacks and 1.3% strokes) and favorable low thirty-day mortality of only 3.9% [23]. The reported outcome events are quite low and advocate the simultaneous approach of CEA and heart surgery. Of note, the reported patient population was selected and may not reflect an allcomers collective. Nevertheless, the discussion of the most optimal surgical approach is ongoing, and the literature clearly shows that patients who require treatment for both significantly carotid and coronary stenosis have a higher risk of comorbidity than those who have either isolated myocardial disease or carotid revascularization [21]. Klarin et al. reported on a comparative study including patients undergoing coronary artery bypass grafting with or without CEA of an internal carotid stenosis with a stenosis grade of at least 80%. The authors stated that there were no differences in postoperative stroke rates or thirty-day all-cause mortality when comparing both groups, with respect to on- and off-pump surgery [24]. On the other hand, Minisandran et al. reported on favorable 30-year experiences with simultaneous CEA in combination with coronary artery bypass grafting and further advocate this approach [25]. In a landmark study from 1996, Roach et al. reported a proximal high-grade aortic atherosclerosis as an independent stroke predictor [26], as athero-arterial embolism from the ascending aorta is believed to be a source of embolic stroke related to aortic manipulation or clamping of the aorta ascendens [27]. Further, in most of the cases a postoperative related stroke after coronary artery bypass grafting occurs later than 24 h after surgery, suggesting that factors unrelated to carotid diseases, such as diabetes mellitus, atrial fibrillation and postoperative blood pressure fluctuations, play an essential role [21]. Many surgeons seem to prefer a simultaneous CEA approach while preceding cardiac surgery as a “combined-sequential” approach; a real-world simultaneous approach is rarely performed for reasons of practicality and hemodynamics. In contrast, Khaitan et al. describe low mortality and stroke rates with a single-stage procedure when carotid endarterectomy is performed after cardiopulmonary bypass under moderate systemic hypothermia used for cerebral protection [28,29]. The authors justify this approach with better rheological conditions and given cerebral protection under moderate hypothermia. Several studies demonstrate some of the difficulties related to this combined disease process. However, they stated that symptomatic status and degree of stenosis are crucial and associated with worse outcomes, also when a stenting is performed [30,31]. The revascularization strategy is also of particular importance. When evaluating the risk, findings show that the combination of CABG and CEA carries low risk and can be performed with acceptable outcomes. 

## 5. Study Limitations

Our study is limited by the retrospective and single-center design and reporting relatively small cohort of patients without an out of hospital follow-up. Hence, the presented data have to be taken with caution and are not calculated for statistically significant outcome measures. Further, we only present an analysis of patients undergoing simultaneous CEA and heart surgery as a comparison to patients undergoing secondary CEA after heart surgery or vice versa are lacking and still an objective of our study group. Further, there is no comparator group with patients undergoing heart surgery and significant internal carotid artery stenosis without carotid surgery or stenting.

## 6. Conclusions

Patients with simultaneous relevant internal carotid artery stenosis who are in need for heart surgery represent a high-risk population as it is critically to discuss which approach has to be addressed first while they show elevated risks for severe perioperative neurological and cardiovascular complications, which can present heterogeneously as shown in this study. Whether a concomitant or staged CEA is the best option for patients with significant internal carotid artery stenosis remains debatable. If performed, simultaneous carotid and heart surgery remain a safe option for this highest-risk patient collective in the hands of experienced surgeons. Within the reported patient population suffering from MACCE after a simultaneous carotid endarterectomy and heart surgery, a preoperative history of transitory ischemic attack and kidney disease might account for worse outcomes, as severe events were not only neurologically driven but also associated with postoperative cardiovascular complications following heart surgical procedures.

## Figures and Tables

**Table 1:** Preoperative patient characteristics.

Preoperative Characteristics	No-MACCE (*n* = 87)	MACCE (*n* = 24)	*p*-Value
Age, years	77 ± 8.3	77 ± 7.2	0.649
Male, *n* (%)	75 (86)	19 (79)	0.521
Height, meter	1.81 ± 0.6	1.82 ± 0.6	0.484
Weight, kilogram	93 ± 4.7	92 ± 5.3	0.380
Body mass index, kg/m^2^	28 ± 1.7	28 ± 1.7	0.079
Smoking history, *n* (%)	53 (61)	15 (63)	0.888
Chronic obstructive pulmonary disease, *n* (%)	3 (3,4)	4 (17)	0.038
Pulmonal hypertension, *n* (%)	3 (3,4)	3 (13)	0.114
Diabetes mellitus, *n* (%)	55 (63)	17(71)	0.489
Hyperlipoproteinemia, *n* (%)	87 (100)	22 (92)	0.045
Arterial hypertension, *n* (%)	86 (99)	23 (96)	0.387
Chronic kidney disease, *n* (%)	11 (13)	8 (33)	0.029
Peripheral arterial disease, *n* (%)	25 (29)	12 (50)	0.050
Coronary artery disease, *n* (%)	86 (98)	22 (91)	0.210
Instable angina pectoris, *n* (%)	5 (5,7)	8 (33)	0.001
Prior myocardial infract, *n* (%)	7 (80)	3 (13)	0.448
Prior coronary intervention, *n* (%)	9 (10)	6 (25)	0.107
Prior percutaneous coronary intervention, *n* (%)	8 (9,2)	6(25)	0.098
Shock/Decompensation, *n* (%)	25 (29)	9 (38)	0.410
Sinus rhythm, *n* (%)	14 (16)	7 (29)	0.154
Left-ventricular ejection fraction <30%, *n* (%)	3 (3.4)	2 (8.3)	0.295
Prior heart surgery, *n* (%)	3 (3.4)	1 (4.2)	1.000
Carotid internal stenosis, grade in %	87 ± 4.5	88 ± 3.0	0.353
Carotid internal stenosis <90%, *n* (%)	44 (51)	9 (38)	0.256
Carotid internal stenosis bilateral, *n* (%)	10 (12)	4 (17)	0.498
Other intracranial stenosis, *n* (%)	1 (1.5)	0 (0.0)	0.597
Prior carotid internal intervention, *n* (%)	4 (4.6)	1 (4.2)	1.000
Neurological disability, *n* (%)	2 (2.3)	0 (0.0)	1.000
Prior stroke, *n* (%)	4 (4.6)	3 (13)	0.171
Prior transitory ischemic attack, *n* (%)	5 (5.7)	5 (21)	0.037
Aspirin, *n* (%)	39 (45)	10 (42)	0.782
P2Y12-Inhibitor, *n* (%)	5 (5.7)	0 (0.0)	0.583
Direct oral anticoagulation, *n* (%)	1 (1.1)	0 (0.0)	1.000
Vitamin K-Antagonist, *n* (%)	7 (80)	5 (21)	0.129
Beta blockers, *n* (%)	68 (78)	20 (83)	0.778
ACE inhibitor, *n* (%)	76 (87)	20 (83)	0.736
AT II inhibitor, *n* (%)	6 (6.9)	4 (17)	0.219
Statin, *n* (%)	72 (83)	21 (88)	0.759

Data is expressed as mean ± standard deviation (SD) or counts (percentage) as indicated. MACCE = Major Adverse Cardiac and Cerebrovascular event.

**Table 2:** Intraoperative characteristics.

Intraoperative Characteristics	No-MACCE (*n* = 87)	MACCE (*n* = 24)	*p*-Value
Carotid endarterectomy with patch, *n* (%)	14 (16)	3 (13)	1.000
Coronary artery bypass grafting, *n* (%)	80 (92)	21 (86)	0.448
Aortic valve replacement, *n* (%)	19 (22)	6 (25)	0.743
Mitral valve replacement, *n* (%)	1 (1.1)	2 (8.3)	0.117
Mitral valve repair, *n* (%)	4 (4.6)	1 (4.2)	1.000
Tricuspid valve repair, *n* (%)	2 (2.3)	1 (4.2)	0.522
Atrial ablation, *n* (%)	3 (3.4)	1 (4.2)	1.000
Atrial exclusion, *n* (%)	5 (5.7)	1 (4.2)	1.000
Concomitant surgery, *n* (%)	17 (20)	7 (29)	0.310
Use of cardio-pulmonal bypass, *n* (%)	80 (92)	22 (92)	1.000
Cardiopulmonary bypass time, minutes	91 ± 33	95 ± 51	0.543
Aortic cross clamp time, minutes	52 ± 20	51 ± 26	0.345
Operation time, minutes	258 ± 55	268 ± 76	0.659
Intra-aortic balloon pump, *n* (%)	1 (1.1)	6 (25)	0.002

Data are expressed as mean ± standard deviation (SD) or counts (percentage) as indicated. MACCE = Major Adverse Cardiac and Cerebrovascular event.

**Table 3:** MACCE characteristics.

Postoperative Characteristics	MACCE (*n* = 24)
Major adverse cardiac and cerebrovascular events, *n* (%)	24 (100)
- Thirty-day all-cause mortality, *n* (%)	7 (29)
- Valve related mortality, *n* (%)	1 (4.2)
- Myocardial infarction, *n* (%)	1 (4.2)
- Stroke, *n* (%)	12 (50)
- Transitory ischemic attack, *n* (%)	6 (25)

Data are expressed counts (percentage) as indicated. MACCE = Major Adverse Cardiac and Cerebrovascular event.

**Table 4:** Postoperative characteristics.

Postoperative Characteristics	No-MACCE (*n* = 87)	MACCE (*n* = 24)	*p*-Value
Major bleeding, *n* (%)	0 (0.0)	9 (38)	<0.001
Re-thoracotomy, *n* (%)	1 (1.1)	8 (33)	<0.001
Cardio-pulmonary reanimation, *n* (%)	0 (0.0)	8 (33)	<0.001
Shockable rhythm disturbances, *n* (%)	22 (25)	14 (58)	0.002
New onset postoperative dialysis, *n* (%)	2 (2.3)	7 (29)	<0.001
Intra-aortic balloon pump, *n* (%)	0 (0.0)	3 (13)	0.009
Extracorporeal membrane oxygenation, *n* (%)	0 (0.0)	1 (4.2)	0.216
Brain computer tomography, *n* (%)	6 (6.9)	14 (58)	<0.001
Symptomatic transitory psychotic syndrome, *n* (%)	34 (39)	12 (50)	0.336
Serious adverse events other than MACCE, *n* (%)	2 (2.3)	4 (17)	0.019
Invasive ventilation duration >12 h, *n* (%)	5 (5.7)	13 (54)	<0.001
Intensive care unit stay, days	4.1 ± 4.8	12 ± 17	<0.001
Overall in hospital stay, days	14 ± 6.5	17 ± 17	0.593

Data are expressed as mean ± standard deviation (SD) or counts (percentage) as indicated. MACCE = Major Adverse Cardiac and Cerebrovascular event.

## Data Availability

Data are available on a special request.
